# The need for smart microalgal bioprospecting

**DOI:** 10.1007/s13659-024-00487-3

**Published:** 2025-01-16

**Authors:** Joan Labara Tirado, Andrei Herdean, Peter J. Ralph

**Affiliations:** Faculty of Science, Climate Change Cluster (C3), Algal Biotechnology & Biosystems, University of Technology Sydney, Sydney, NSW 2007 Australia

**Keywords:** Microalgae, Bioprospecting, Fluorescent probing

## Abstract

Microalgae’s adaptability and resilience to Earth’s diverse environments have evolved these photosynthetic microorganisms into a biotechnological source of industrially relevant physiological functions and biometabolites. Despite this, microalgae-based industries only exploit a handful of species. This lack of biodiversity hinders the expansion of the microalgal industry. Microalgal bioprospecting, searching for novel biological algal resources with new properties, remains a low throughput and time-consuming endeavour due to inefficient workflows that rely on non-selective sampling, monoalgal culture status and outdated, non-standardized characterization techniques. This review will highlight the importance of microalgal bioprospecting and critically explore commonly employed methodologies. We will also explore current advances driving the next generation of smart algal bioprospecting focusing on novel workflows and transdisciplinary methodologies with the potential to enable high-throughput microalgal biodiscoveries. Images adapted from (Addicted04 in Wikipedia File: Australia on the globe (Australia centered).svg. 2014.; Jin et al. in ACS Appl Bio Mater 4:5080–5089, 2021; Kim et al. in Microchim Acta 189:88, 2022; Tony et al. in Lab on a Chip 15, 19:3810–3810; Thermo Fisher Scientific INC. in CTS Rotea Brochure).

**Graphical abstract:**

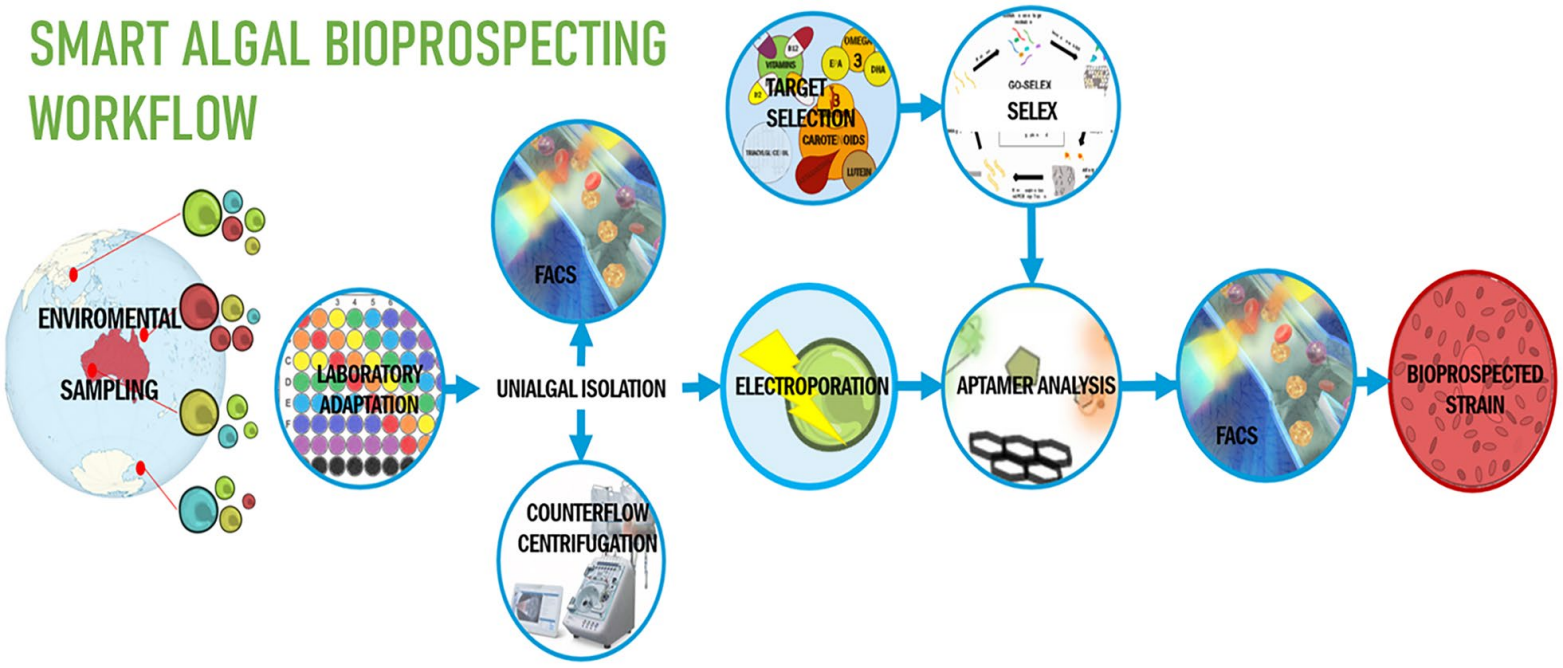

## Introduction

In recent years, the CO_2_ emission and climate change-induced necessity of developing new Green Circular Economies and CO_2_ fixation technologies has put microalgae in the spotlight [6, 7]. Microalgae, a term used to describe a biodiverse polyphyletic group comprised of both eukaryotic (*Glaucophyta, Rhodophyta, Chlorophyta, Haptophyta, Cryptophyta, Ochrophyta, Miozoa, Cercozoa* and *Euglenozoa*) and prokaryotic (*Cyanobacteria*) taxa [8, 9], are predominantly photosynthetic microorganisms [7, 10–12] which can be ubiquitously found across all of Earth’s habitats [7].

Microalgae's valuable biochemical composition, photosynthetic and CO_2_ fixation efficiency, fast growth rates and their lack of competition for arable land have spawned the creation of novel microalgal industries such as biomanufacturing, atmospheric carbon capture and its conversion into high-value bioproducts and/or biofuels [6, 7, 13]. Despite microalgae's biodiversity [8, 9], these nascent industries only employ a limited number of species, restricting their abilities to exploit the diverse biological properties present across the wide range of microalgal taxa [14–16].

Bioprospecting, the systematic search for novel microalgae with biotechnological and/or commercial potential, is an essential requirement for the expansion of the microalgal industry [17]. However, current microalgal bioprospecting suffers from low-throughput, inefficient, time-consuming and non-scalable methodologies. This review aims to provide an updated overview of the current state of microalgal bioprospecting, addressing its limitations and pitfalls. By providing a comprehensive perspective on currently used methodologies, identifying and critically analysing innovative and transdisciplinary approaches, this review aims to serve as a source of inspiration for advancing the implementation of smart microalgal bioprospecting.

## Climate change: a substrate for a microalgal revolution

### Global carbon engineers

In the late Archean Eon, *Cyanobacteria* pioneered oxygenic photosynthesis, culminating in the Great Oxidation Event that transformed Earth's atmosphere and facilitated the evolution of aerobic life [18, 19]. Microalgae have successfully colonized Earth’s photic habitats [20–26]. Adapting to this broad habitat range has exposed microalgae to diverse selective pressures, fostering their remarkable phenotypic and ecological diversity [12]. Regardless of their versatility, all microalgae play a pivotal role in global nutrient cycling [12, 27]. Microalgae annually contribute 50% of the total globally assimilated carbon [28] and drive the biological carbon pump [29] responsible for the long-term storage of carbon in the deep ocean [30]. As primary producers, microalgae form the basis of aquatic food webs [30] and are responsible for the biosynthesis of essential biomolecules such as Docosahexaenoic Acid (DHA), the bioaccumulation of which across higher trophic levels led to early human brain evolution [31, 32].

Like the rest of Earth’s species, microalgae are threatened by climate change [33] driven by anthropogenic CO_2_ emissions [34]. The eventual translation of global warming consequences into ocean ecosystems will reduce marine CO_2_ availability due to ocean acidification and salinification [30]. These environmental shifts will pose complex challenges to microalgae, potentially altering their physiology, ecological interactions, and community compositions, which may cascade to higher trophic levels and devastatingly disrupt ecological and geochemical processes [28, 30, 33, 35].

### Microalgal cell factories for every industry

**Fig. 1:**
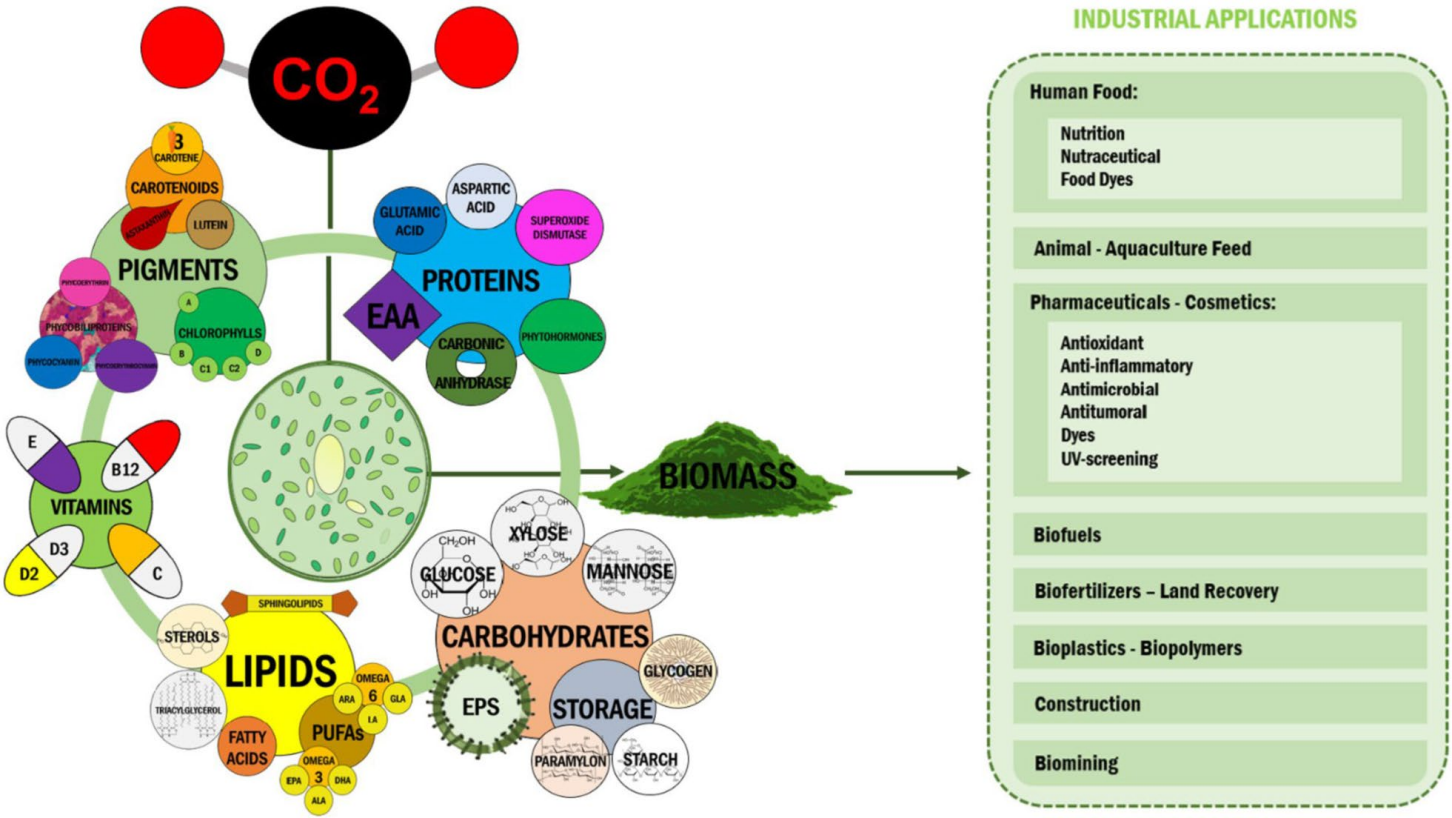
Microalgal carbon capture and manufacture (CCM) [43, 47–50]. Microalgae’s efficient CO_2_ biofixation, carbon conversion and long-term storage into high-value biometabolites offers the grounds for developing products and services for an economically profitable, green and circular economy

*Chlorella vulgaris*, isolated and studied in 1890, marked the beginning of scientific microalgal exploration [36–38]. However, human-microalgae interactions date back centuries, for instance, the consumption of *Arthospira* (*Spirulina*) can be traced back to the fourteenth century [39, 40]. Microalgae have shaped human evolution [31, 32], but they might also allow us to evade the CO_2_ emission-driven path of climate change-led collapse that the industrially developed world is currently following [34, 41, 42]. International efforts now strive for carbon neutrality [42, 43], an endeavour necessitating innovative biotechnological technologies [44–46] and, as in the Archean Eon [18], CO_2_ has again put microalgae in a central position. Microalgae are considered a CO_2_ mitigation strategy [7, 47] due to their rapid growth and CO_2_ fixation rates, not requiring arable land for cultivation and being a cellular factory of biotechnologically valuable carbon molecules [47–49] (Fig. 1).

## Microalgal bioprospecting

### Bioprospecting microalgae's biodiversity dark matter

Microalgae support a diverse array of industries [16]. Regardless of microalgae's high biodiversity [9], only a limited number of microalgal species are currently exploited in industrial processes [15, 16, 51–54] (Table 1).

**Table 1:** Microalgae taxa with commercial applications, adapted from [15, 53, 54]

Microalgae	Product	Application
*Chlorella vulgaris*	Biomass, Carbohydrates, Vitamin C	Health food, food supplement, animal feed
*Chlorella* spp*.*	Biomass, Carbohydrates, Vitamin C	Health food, food supplement, animal feed, wastewater treatment
*Arthospira platensis*	Biomass, Protein, γ-Linolenic Acid, Vitamin B_12_, β-carotene, Phycocyanin	Health food, food supplement, pharmaceutical, cosmetics, animal feed
*Arthospira* spp*.*	Protein, γ-Linolenic Acid, Vitamin B_12_	Health food, food supplement, animal feed
*Dunaliella salina*	β-carotene, Carotenoids	Health food, food supplement
*Haematococcus pluvialis*	Astaxanthin, Carotenoids	Health food, pharmaceuticals
*Nostoc* spp*.*	Biomass, PUFA, Immune modulators, Antimicrobial extracts, Anticancer extracts, Phycocyanin	Health food, food supplement, pharmaceutical, biofertilizers

Described microalgal biodiversity encompasses 50,000 species [55], with conservative estimates placing total diversity at around 200,000 species [56]. Despite this high biodiversity, the number of commercially cultivated species remains in the dozens, with highly economically developed regions like the European Union only cultivating 46 microalgal species [51]. This under-utilization of microalgal biodiversity constrains the nascent industry, impedes diversification into novel applications and reduces industrial resilience.

**Fig. 2:**
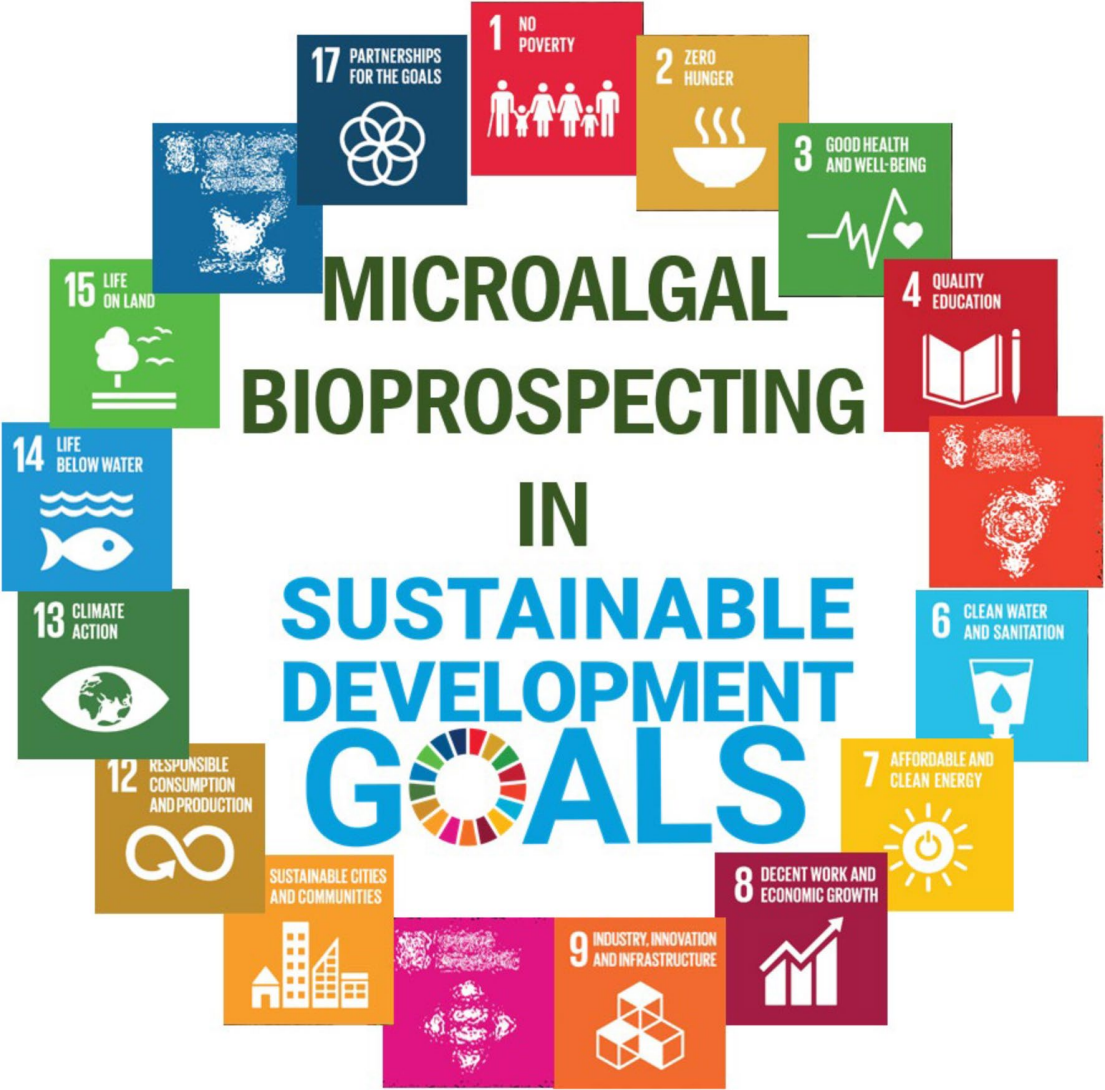
Microalgal bioprospecting aids in the accomplishment of 14/17 UN SDGs* [58, 59]. *The content of this publication has not been approved by the United Nations and does not reflect the views of the United Nations or its officials or Member States

Bioprospecting, systematically sampling and selecting nature's biological resources—taxonomic species, biomolecules, biosynthetic pathways, genes or genomes—offers the possibility of harnessing microalgal biodiversity by exploring novel microalgae. Leveraging microalgae's sustainable nature, bioprospecting not only supports industrial, commercial or research microalgal applications [57], but also aligns with the accomplishment of several United Nations Sustainable Development Goals (UN SDGs) [58] (Fig. 2). This presents a new opportunity for discovering novel species, biometabolites and genes using novel biotechnological methodologies to exploit microalgae's untapped potential.

### Current state of microalgal bioprospecting

Bioprospecting, like microalgal consumption as a food source [39, 40], has been omnipresent throughout human history [60]. The expansion of the commercial use of microalgae beyond the food industry, sparked by the US oil crises in the latter half of twentieth century [61], propelled large-scale algal cultivation and intensified microalgal bioprospecting. This, in conjunction with academic advances, has led to the discovery and characterization of diverse taxonomical alternatives to develop the ever-widening microalgae-supported industrial applications.

Microalgal bioprospecting methodologies involve two distinct phases: [1] microalgae sampling and isolation and [2] characterization to select microalgae with desired traits. Workflows can be classified further into univariate approaches, limited to widespread sampling and characterization for a specific trait, and multivariate methodologies that integrate multiple selection strategies in the search of strains combining several traits to narrow down the pool of possible candidate taxa [62].

#### Sampling and isolation

The first step in bioprospecting is sampling, which is typically achieved by directly collecting water samples in aquatic environments. Despite this, many other available methodologies have been adapted to non-aquatic sampling [63–65]. Upon arrival at the laboratory, samples generally undergo nutrient enrichment with different media; typically, ES, F/2 or BG11SW, to promote specific taxa [63]. Subsequently, single-cell or agar isolation methodologies, often combined with dilution techniques, isolate specific microalgae to establish mono-algal cultures [63]. Depending on factors like sampling location, identified microalgal taxa, and intended use of the culture, axenic cultures may be obtained through selective treatments with antimicrobials such as antibiotics, antifungals, antivirals, and/or enzymatic treatments [63].

#### Traditional characterization methodologies

Varied characterization methodologies can be applied depending on the methodology and the desired phenotypic trait/s, an observable and quantifiable characteristic/s, used for selection. Contrarily, growth rates, commonly estimated from correlation between Optical Density (OD) measurements with a spectrophotometer; typically at 680–750 nm [66–68], and cell counts; performed with a haemocytometer chamber [69, 70], are a point of consensus across microalgal bioprospecting studies (Table 2). Growth kinetics can also be employed in a multivariate manner [66, 69, 71–73]. For instance, Rezaei, et al. (2023) simultaneously isolated, grew and selected high growing strains bioprospected from high-mountain lakes and grown in cold conditions [66] and Condori, et al. (2024) bioprospected microalgae from contaminated water environments to characterize their growth and nutrient removal efficiency when grown in explosive industry wastewater [69]. These manifold combinations of screening criteria enable the selection of algal strains both successfully and rapidly growing under a selected set of stringent culture conditions [66, 69, 71, 73]. Similarly prevalent is the weighting of dry biomass; performed by sun, hot air, oven, spray or freeze drying [68, 70, 74, 75].

**Table 2:** Commonly employed characterization methodologies in microalgal bioprospecting

Target Trait	Methodology	Throughput	Example of application
Growth, biomass and photosynthetic metabolism			
Growth kinetics	Optical density (OD)	High	[66–68]
	Cell Counts	Low	[69, 70]
Biomass weight	Dry biomass weighting	Low	[68, 70, 74]
Growth under specific conditions	Selection	Medium–High	[66, 69, 71–73]
Biometabolite characterization			
Lipids and fatty acids	Gravimetric analysis	Low	[66, 69, 74]
	Chromatography	Low	[68, 76]
Pigments	OD and equations	Low	[67–69, 84]
	Chromatography	Low	[70, 78]
Carbohydrates	Dubois phenol–sulfuric acid method	Low	[67, 69, 74, 86]
	Chromatography	Low	[88, 89]
Protein	Lowry method	Low	[70, 74]
	Coomasie blue dye staining	Medium–High	[67]
	Nitrogen-to-protein conversion rate	High	[69, 89]

Microalgae's taxonomic biodiversity has turned these microorganisms into cellular powerhouses to produce an even more diverse range of biometabolites [47–49]. As such, biometabolite characterization; the identification, study and quantification of the industrially relevant molecules present in newly bioprospected isolates, is a common step in microalgal bioprospecting workflows.

Bioprospecting efforts in microalgae, driven by the pursuit of economically viable biofuels, have consistently focused on lipid and fatty acid characterization [7, 66, 68–70, 74, 76–78]. Traditionally, multi-step gravimetric methods have been employed to quantify lipid fractions, known for their precision but limited in providing a comprehensive lipidomic profile [66, 69, 74, 78–80]. Alternatively, chromatographic techniques such as Thin-Layer (TLC), High-Performance Liquid (HPLC), or Gas Chromatography (GC) [66, 68, 70, 76] [79] coupled with Mass Spectroscopy (MS), Flame Ionization Detector (FID) or Time-Of-Flight (TOF) [79, 81] offer detailed analyses of transesterified or transmethylated fatty acids [81], enhancing the effectiveness of the lipidomic assessment for biofuel potential [85, 87]. This is the case of Ammar, et al. (2024), which simultaneously gravimetrically quantified and GC–MS profiled the lipid and Fatty Acid Methyl Ester (FAME) fractions of bioprospected Tunisian algal isolates [68].

Photosynthetic microalgal pigments—chlorophylls, carotenoids, and phycobilins—are pivotal in microalgal biometabolite production, attracting significant bioprospecting interest [82]. Pigments are analysed after having been extracted using organic solvents [83] through spectrophotometric readings at pigment-specific autofluorescence wavelengths, often using equations to extrapolate these reads for total pigment quantification [67–69, 84]. Variations include HPLC-based quantification as seen in Grubišic, et al. (2022) or Patel, et al. (2022), who also extrapolated the total pigment content of their bioprospected microalgae by using established equations [70, 78].

Due to their diverse applications [47, 49, 85] microalgal carbohydrates are also repeatedly bioprospected [67, 69, 74, 86]. Commonly, carbohydrate quantification is performed through the phenol–sulfuric acid method by Dubois, et al. (1956) [87] or similar derivatives [86, 88]. However, just as with gravimetric lipid quantification, Dubois and derivative methods do not explore the carbohydrate profile; a knowledge gap also solved by GC–MS or GC-FID [88, 89].

Microalgal proteins; yet another highly sought after algal biometabolite [90, 91], are quantified using spectrophotometric methodologies [67, 70, 74]. Both Assobhi, et al. (2024) and Grubišic, et al. (2022) employed the Lowry method [92] for protein quantification [70, 74], a technique based on colorimetric change [93]. Comparably, Araj-Shirvani, et al. (2024) read the OD at 595 nm of their Coomassie Blue Dye stained sample and compared the results with a bovine serum albumin standard [67]. A different approach can be found on Condori, et al. (2024) [69] or Cruz, et al. (2023) [89], both of which indirectly quantified protein content by using a nitrogen-to-protein conversion rate obtained from the literature [94].

Notably, microalgal bioprospecting efforts span far beyond major metabolite classes and include singular metabolites such as phenolic compounds [67, 70, 95], flavonoids [70], and lipid, protein or carbohydrate extracts for their potential applications as antimicrobial [70, 96], anticancer [95, 97], antioxidant [67, 70, 95, 98], anti-ageing [95], enzyme inhibiting [95] or prebiotic agents [95]. Additionally, physiological algal processes like bioaccumulation [69, 99, 100], biomineralization [101] phytohormone [102], exopolysaccharide (EPS) [102, 103] or nanoparticle (NP) [104] production are also bioprospected for their industrial applications.

### Bioprospecting: a Herculean endeavour

Despite significant scientific and technological advancements in marine biotechnology [105], the continuous worldwide expansion of the microalgal industry [16] and the rapid development of marine bioprospecting [106], microalgal bioprospecting remains a burdensome endeavour.

To begin with, environmental sampling methodologies are fast and inexpensive [63], but developing a gold-standard guide for best microalgal sampling practice is still to occur [107]. Moreover, sampling location and sample quantities are often determined by convenience, geographic proximity and the presence of a water body. This non-selective bioprospecting overlooks the ecological evolution and natural selection history that shape stress-adapted microalgal biometabolite composition, disregarding the invaluable natural linkage between desired phenotypes and sampling locations [108]. Indiscriminate sampling may yield diverse samples, but technical constraints in post-sampling isolation, culturing, and biometabolite characterization make this approach inefficient. The primary obstacle for bioprospecting pipelines is the current requirement of monoalgal status, a culture condition only achieved after a time-consuming and resource-intensive separation process. Additionally, improper enrichment and isolation workflows can arise taxonomic prevalence and survival biases stemming from enrichment-media selection, microbial competition or, in the case of dinoflagellate presence, to outright predation [109, 110]. Adding to this low-throughput wound is the requirement, a recurrently left desire, for axenic cultures in most current microalgal characterization methodologies [111]. Furthermore, a full methodology guide for the achievement of this tedious, time-consuming and hardly achievable; or impossible [112] axenic outcome is also yet to be created [63].

Similarly ineffective, current microalgal characterization methodologies require a biomass yield in the milligrams [107]. Ideally, this excruciating high biomass yield should be achieved with culture optimization but realistically, bioprospecting efforts do not progress beyond basic discriminations such as fresh or saltwater [70, 84, 86]. This presents a double-edged dilemma as, while rapid growth is a desirable trait, its relevance is diminished if the culture conditions used for its assessment do not match the future culturing parameters required at the prospective application of the strain. Furthermore, the induction of several commonly characterized phenotypic traits; such as lipid production, is largely culture-parameter-dependent [113]. Consequently, a lack of consideration for phenomic plasticity, the ability of an organism to adapt its phenotypic traits to the culture conditions its subject to [114], and this linkage between culture conditions and observable phenes, can result in the erroneous selection of inefficient microalgal isolates or the overlooking of the most efficient ones.

Likewise, the number of desired phenotypic traits and the methodologies employed for their characterization are crucial. Yet, commonly employed characterization methodologies are outdated, low throughput and fail to characterize all relevant parameters [115]. The former and the latter can be exemplified by gravimetric [66, 69, 74] or phenol–sulfuric quantification [67, 69, 74, 86] methodologies. These multi-step, tedious processes requiring milligram quantities of biomass are, on top of that, unable to profile the lipid and carbohydrate species present in the samples respectively [79, 80, 115]. Though they are an improvement, chromatographic techniques are not the silver bullet of microalgal characterization due to their multistep, time-consuming and costly nature, their unforgiving sample quantity requirements and their difficult result standardization across studies [116].

Overall, outdated, inefficient and low throughput culturing, isolation and characterization methodologies (Table 2) [115] compromise the application of efficient workflows and are significant hurdles to the development, application and economical success of microalgal bioprospecting and the expansion of the algal industry.

## Smart microalgal bioprospecting

Microalgal bioprospecting is currently limited by the employment of low throughput methodologies, a subsequent impediment to the implementation of a biodiverse portfolio of industrially profitable microalgae species. To respond to this impending need, we propose the development of a new wave of high-throughput ''smart'' microalgal bioprospecting workflows founded on ecological considerations, novel approaches and robust methodologies, equipment and techniques.

### From the pond to the laboratory

Sampling and targeting everything, then selecting later is not currently viable. Therefore, smart bioprospecting must aim to enhance the likelihood of isolating only desired microalgal isolates. This must begin with a critical assessment of environmental sampling locations with ecological pressures that are likely to generate the desired microalgal phenotypic traits [17, 62], an approach known as bio-rational collection and screening [17, 62]. Similarly desirable is the application of multivariate approaches that link bio-rational sampling with natural-pressure-imitating stringent laboratory culturing conditions [66, 69, 71, 73]. Combined, these methodologies significantly decrease the pool of ''to-be-characterized'' microalgal strains [62], lowering economic, human and time costs [63] and enhancing throughput. The success of this is exemplified by Royal DSM, a Dutch company that has achieved commercialization and EU novel food approval for a high DHA-producing *Schizochytrium sp*. strain bioprospected bio-rationally [17, 117, 118].

Despite marketing microalgae's biodiversity, many “bioprospecting” efforts limit their activity to characterizing strains from culture collections [17]. Despite being a source of potentially relevant strains [119], culture collections do not sample bio-rationally. As such, indiscriminate ''culture collection bioprospecting''; coupled with the likely genetic drift, shifts in allele frequencies arisen during population bottlenecks generated by routine serial subculture, and phenotypic changes that a long-term culture is prone to suffer [120, 121], is likely to yield non-competitive strains. Additionally, climatically and microbially ecological obsolescence makes this approach suboptimal when prospective large-scale culturing is a desired bioprospected strain trait. Conversely, local bio-rational sampling offers access to ecologically-competitive isolates [122, 123]. Nevertheless, exotic sampling also holds vast potential. For instance, targeting extremophilic microalgae capable of thriving under cultivation conditions lethal to contaminants is a proven commercially successful approach, as evidenced by *Dunaliella salina* or *Arthospira platensis* dominating their respective markets [24, 124].

Combining bio-rational sampling with multivariate approaches and focusing on unique natural [125–127] or man-made [128, 129] ecological niches will enhance both the taxonomical diversity of industrially exploited microalgal species and the discovery of novel compounds with industrial applications [130–132].

### From algal soup to monoalgal culture

Bettering the currently infuriatingly low-throughput enrichment and monoalgal culture isolation [133] requires adaptability to the objectives behind smart bioprospecting. For instance, approaches desiring taxonomical biodiversity should focus on impeding the introduction of unwanted biases. This can be achieved by the fractionation of the original multialgal sample into smaller individual aliquots followed by the enrichment of each individual fraction with multiple distinct culturing medias and conditions. In turn, these differing parameters foster aliquot-specific “biases”, which result in differing taxa-specific growth and, even, survival rates. On the contrary, multivariate approaches used to isolate growth under stringent wastewater [134], salinity [135], pH [136, 137], CO_2_ [138] and light [139] parameters should continue their expansion towards novel selecting parameters such as growth in anaerobic digestates [140] or unfiltered coal-fired flue gas [141].

Regardless, implementing automation and high-throughput cultivation methodologies are increasingly imperative. Multi-well plates [142, 143] and microfluidic platforms [144–146] offer throughputs orders of magnitude above traditional culturing, both in terms of time and in the number of nutrient profiles tested per run [145, 147]. An example is provided in Radzun, et al. (2015) which rapidly optimized the composition and individual concentrations of 12 macro- and microelements under non-limiting CO_2_ and light conditions in an automated 1,728 multi-well setup considering the maximum growth rates of 8 different microalgae [147]. Despite these advances, workflow limitations persist due to the small volumes offered by these approaches. Overcoming this also requires automation, which is now implemented in photobioreactors to provide larger culturing volumes. This nascent field, practically unknown to microalgal bioprospecting, is highly adaptable and allows the monitoring of as many culture parameters as sensors exist [148–151] and, as seen in Nguyen, et al. (2018) [150], the adoption of cheap sensor-culturing setups will reduce processing time and costs, reallocating resources to other crucial non-automatable processes.

Nevertheless, no recent technical advances have been as impactful and widely adopted in microalgal bioprospecting as Flow cytometry (FC) and Fluorescence-activated cell sorting (FACS). FC offers rapid and reliable screens of environmental samples through single-cell interrogation by laser interception and detection of scatter light to generate datasets with ''events'' that represent different cell populations. Coupled with a cell sorter, FC becomes FACS, enabling the isolation of desired populations based on specific sets of parameters called "gates". This technology, as demonstrated by Jakob's, et al. (2013) [152], has no less than revolutionised microalgal isolation into monoalgal cultures [63, 133, 153]. FACS is also instrumental in generating monoalgal axenic cultures [63, 152–154]. By interrogating environmental samples for chlorophyll fluorescence, it distinguishes chlorophyll-containing algal cells from bacteria, dead cells, and debris, after which, chlorophyll-positive gating allows monoalgal isolation [111, 152, 155]. Despite its benefits, FACS isolation exposes cells to shear force, electrostatic charges and high-energy lasers. As also seen in Jakob’s, et al. (2013), the success of FACS depends on cell parameters like size, shape, abundance and hardiness [152, 154], limiting its universal applicability across all microalgae taxa [63, 154]. This prevents abandoning micromanipulation, the isolation of single cells through aspiration with microcapillaries, a resource-intensive, low-throughput technique requiring highly skilled microscopy users proficient in microalgal morphological identification.

However, an evolution in centrifugation technology holds promise, whereas FACS has limitations. Traditional centrifugation is integral in microalgal studies for tasks like supernatant removal, re-culturing and biomass harvesting [156]. Although density centrifugation has also been proposed to achieve axenic cultures [157], it requires optimization, lacks automation and can reduce cell viability due to shear forces [156]. A promising high-throughput and automatable alternative lies in counterflow centrifugation-based instrumentation [158]. Widely used in cell therapy [158, 159] for its cell concentration, buffer exchange and cell-size based separation capabilities with minimal impact on cell viability [160], the potential of counterflow-centrifugation is yet to be investigated by microalgal researchers. Therefore, counterflow centrifugation could greatly benefit microalgal bioprospecting by enabling size-based separation among various microalgal taxa and their common contaminants [161], facilitating monoalgal axenic isolation.

Regardless, the taxa-wide prevalence of microalgae-attached bacteria [162] makes neither FACS nor counterflow centrifugation an axenic-obtaining silver bullet. Importantly, the reasons and viability behind removing this unique phycosphere must be addressed as, in some cases, this bacterial ''contamination'' is not only not problematic but essential or positive for microalgal development [162, 163]. This is so much so that phycosphere-bioprospecting for the enhanced production of microalgal-bacterial bioproducts or co-cultivation-arisen microalgal productivity improvements is an increasingly developing field [164–167]. Despite this, some workflows still require absolute axenic status and, for that, the employment of ultrasonication; > 20 kHz ultrasonic waves, and its high throughput adaption is needed [155].

After establishing monoalgal cultures, the last pre-characterization step is microalgal identification. Both morphological and traditional molecular identification [168] are not conducive to high-throughput applications. While metabarcoding is preferred for its ability to reduce human error and bias, its independence from taxonomic expertise [168], and its capability to comprehensively assess axenic status or identify phycosphere constituents, there remains a need for improved throughput. As such, the implementation of reliable Artificial Intelligence (AI)—Deep Learning Image Recognition software [168–170] or novel molecular identification technologies [170, 171] is necessary. An example of the latter is Jahn's, et al. (2014) development of a high-throughput metabarcoding 12-well plate setup, employing boiled MiliQ water for algal lysis and an automated sequencing chromatogram analysis methodology [171].

### Bioprospecting into the future

Currently, microalgal characterization methodologies are inefficient, have low throughput [115], and they often require monoalgal cultures. Despite the throughput capabilities of existing and proposed methodologies for achieving this unialgal, axenic or not, state, creating and maintaining multiple monoalgal cultures during bioprospecting is nonsensical, given that most will be discarded during characterization. Could selection be achieved from an enriched multi-taxonomical algal mixture? Moreover, what if a living algal sample were not required?

Culture-independent bioprospecting, has been achieved through metagenomic approaches such as Whole Genome Shotgun [172, 173]. Advances in Next Generation Sequencing (NGS) and global sequencing projects have transformed metagenomic mining, enabling the in silico search for genes encoding biometabolites [172], which becomes commercially viable upon successful gene expression [174]. Nevertheless, microalgal genome sequence databases are currently restricted to a small number of model species, and there is insufficient knowledge of microalgal metabolic pathways [175] and achieving reliable genetic transformation of multiple microalgae species remains unattainable [176]. Together, these current factors limit the application of non-culture dependent microalgal bioprospecting.

Spectroscopic imaging techniques have frequently been proposed as alternative culture-dependent characterization methodologies that enable high-throughput, non-invasive and low-cost characterization [177]. Among these, visible/near infrared, Fourier transform infrared (FTIR), and Raman spectroscopy have garnered significant attention due to their potential in microalgal biometabolite characterization. However, these techniques face several limitations that necessitate extensive optimization and complicate their implementation into high-throughput phenotyping workflows [114]. For instance, Raman spectroscopy requires species-specific optimization [114] and is affected by the background fluorescence of microalgal pigments, while FTIR is affected by spectral interference from water [178]. Altogether, and despite their potential, spectroscopic methodologies are not yet well-suited for high-throughput biometabolite and taxa-wide microalgal bioprospecting.

Another proposed high-throughput approach is Fluorescent Probing (FP), the interrogation of autofluorescing molecules or molecule-specific fluorophores to identify and quantify target metabolites [179]. This approach allows for the assessment of non-monoalgal cultures and has been widely used in lipid bioprospecting, with Nile Red and BODIPY 505/515 being the most common fluorophores for cheap and rapid in situ lipidomic assessment [179, 180]. FP provides throughput levels unimaginable with traditional lipid quantifications when integrated with FACS [180] or microplate workflows [181]. Moreover, various other fluorescent dyes, such as SYTOX Green, a nucleic acid binding fluorophore [182, 183], have extended FP far beyond lipidomics [180, 184, 185]. Although FP presents significant potential for enhancing bioprospecting throughput, challenges persist, including variable fluorophore permeation across different taxa [186], interference with microalgal pigment autofluorescence [180] and the need to establish accurate correlations between fluorescence intensity and target molecule quantity. Investigating novel fluorophores, such as AC-202; a promising alternative to BODIPY [187], could help overcome these challenges and facilitate the transition from traditional low-throughput methodologies [179, 186].

Many of the complications associated with fluorophore-based FP can be mitigated by utilizing microalgae's innate ''fluorophores'': pigments. Pulse Amplitude Modulated (PAM) fluorometry [188], is a non-invasive and high-throughput methodology that offers valuable phenotypic insights by assessing light absorption and photoprotective potentials of microalgal strains [189, 190]. Combining PAM with the optimization of cultivation or stress-induction conditions provides a deeper understanding of the interactions between microalgal photophysiology and cultivation parameters [114, 189, 191]. The Phenoplate, Herdean, et al. (2022), exemplifies the high-throughput capabilities of PAM when integrated with variable cultivation parameters [189, 192], suggesting that further development of similar [142] rapid FP multiparametric workflows could revolutionize microalgal characterization.

Nonetheless, current FP is significantly limited by variable cell wall penetrating abilities and the lack of a diverse array of biometabolite-specific fluorophores or probing methodologies. However, a transformative shift towards nucleic acid or peptide-led and electrophoresis or nanoparticle-assisted methodologies appears imminent. Described elsewhere as biosensors [193], these novel metabolite-specific platforms are not merely aspirational but are already being successfully applied in microalgal research [2, 3, 194, 195]. Central to this approach are Aptamers, small DNA, RNA or peptide chains that specifically bind to biomolecular targets. The Systematic Evolution of Ligands by Exponential Enrichment (SELEX) has evolved into a highly adaptable in vivo or in vitro methodology for aptamer synthesis [196]. SELEX can be performed for a myriad of molecular; or cellular targets [196]. Furthermore, the affordable and versatile nature of nucleic acid and peptide modification facilitates post-SELEX enhancements such as truncation, extension, site-directed mutagenesis and modification or attachment of fluorophores [197] and Quantum Dots, novel inorganic fluorophores [198–201].

Widely applied in the biomedical field [202], aptamer-sensing has been primarily applied in microalgae to detect Harmful Algal Bloom-produced biotoxins [203]. Due to their sensitivity, specific binding, rapid and cost-effective development, and post-development adaptability, aptamers are promising for microalgal metabolite sensing. Several research groups have already proved this potential. For example, Prof. Yoon-E Choi's laboratory (Korea University) has successfully employed single-stranded DNA aptamers for the in vivo sensing of ATP, paramylon and, importantly, β-carotene in the non-cell wall possessing microalgae *Euglena gracilis* and *Ochromonas danica* [2, 3, 195, 204, 205]. Furthermore, aptamers targeting biotechnologically relevant molecules, such as the bio-available form of vitamin B12, methylcobalamin [206], and H_2_O_2_, a marker of oxidative stress [207], or specific binding sites, such as the plastoquinone binding niche of Photosystem II D1 protein [208] have been developed and are poised for implementation across a broader spectrum of microalgae. Aptamer designs targeting biological docking sites, organelle-specific motifs, or even cell-specific motifs [209], represent promising avenues for expanding aptamer applications in diverse biotechnological fields and for enabling smart bioprospecting approaches.

Microalgae's cell walls, cell membranes, and organelle walls still pose significant challenges for intracellular aptamer delivery. Common methods for introducing foreign DNA into microalgae include glass bead agitation, microparticle bombardment, *Agrobacterium*-based delivery and bacterial conjugation [210, 211]. However, these techniques are not suitable for high-throughput bioprospecting workflows due to their variable efficiency and, in the cases of *Agrobacterium* delivery or bacterial conjugation [210–212], their non-transient delivery falls under Genetically Modified Organism legislation which would restrict the commercialization of newly bioprospected strains [213]. Despite these challenges, novel intracellular delivery methods are under development [211, 214, 215]. Cell-penetrating peptides, Liposome-mediated delivery and Nanoparticles show promise as alternatives [211], though these emerging approaches require further research to assess their potential, especially with algae. However, even if these methods achieve higher efficiencies, microalgal biodiversity is likely to impede taxa-wide standardized permeation capabilities, a prospective bias that complicates the implementation of these delivery methods for bioprospecting.

In contrast, reversible electroporation, the transient electro-generation of temporary membrane pores, is a reliable and widely used method with a proven record of successful intracellular delivery rates across various microalgae taxa [211, 216]. Electroporation is commonly achieved through Pulsed Electric Fields (PEF), electrical pulses of a voltage in the kV and of a duration in the ms or ns range [217], parameters that require species-specific optimization [216]. Although various methodologies for optimizing electroporation settings exist [216, 218], all electroporation optimization requires monoalgal cultures. Cell size, a parameter that can be selectively managed through upstream methodologies like FACS or counterflow centrifugation, significantly impacts the success of cell permeabilization [219, 220]. Therefore, size-based fractionation of a non-monoalgal sample and its distribution across different electroporation parameter gradients may be essential for achieving efficient taxa-wide intracellular delivery. This approach could be facilitated by commercially available high-throughput, multi-well electroporation platforms or by developing custom in-house setups [221, 222].

Regardless, free-floating aptamers present in a microalgal cytosol would face enzymatic degradation even if successful intracellular delivery is achieved. A solution for this issue is NP conjugation. For example, Prof. Yoon-E Choi's successful aptamer-sensing has been performed through conjugation with Graphene Oxide NPs (GOnS) and Gold NPs (AuNPs) [2, 3, 195, 204, 205]. Interestingly, GOnS provides fluorophore quenching [2, 3, 195, 204, 205], providing a simple yet effective ON–OFF detection platform. Free-floating aptamer-fluorophores are firstly incubated with GOnS to achieve the quenching or OFF state and only after exposure to the aptamer-specific molecule these aptamer-fluorophore complexes detach, subsequently freeing the fluorophore from quenching and achieving the ON state. Furthermore, specific NP designs can also selectively target certain intracellular compartments [223, 224], a transport choice that can also be achieved through NP conjugation with intracellular guiding peptides [225, 226].

## Conclusion

Microalgal bioprospecting holds transformative potential for advancing diverse microalgal industrial applications and bettering industrial robustness. Yet, current efforts remain hindered by a lack of bio-rational sampling and outdated, low-throughput characterization methodologies. Addressing these gaps demands a shift towards Smart Microalgal Bioprospecting.

The impending abandonment of non-selective sampling requires the integration of bio-rational sampling with expanded use of high-throughput tools such as FACS or implementing novel transdisciplinary approaches such as counterflow centrifugation. In combination with the use of automatable high throughput cultivation platforms such as multi-well plates and microfluidics, this new wave of sampling and laboratory adaptation workflows can improve throughputs, reduce costs and facilitate monoalgal isolation at a scale, an essential stage for accurate microalgal characterization. Moreover, emerging technologies such as fluorescent probing, whether non-invasive PAM or novel biosensor platforms are promising in enabling biometabolite detection. Despite the potential, a further array of biometabolite-specific aptamer or peptide-based biosensors must be developed. Furthermore, cell-penetrating peptides, liposome-mediated delivery, nanoparticles and electroporation technologies require further development to achieve taxa-wide intracellular biosensor delivery.

In addressing these current challenges and exploring emerging technologies with bioprospecting potential, this review aims to aid fellow researchers in the rethinking, developing and implementing of a new wave of smart microalgal bioprospecting efforts.

## Data Availability

No data was used for the research described in the article.
